# AI-Augmented Digital Diagnostics in Minimally Invasive Dentistry: A Case Report on Clinically Occult Approximal Caries

**DOI:** 10.3390/healthcare14193282

**Published:** 2026-10-02

**Authors:** Michal Augustín, Tomáš Siebert

**Affiliations:** 1 Department of Stomatology and Maxillofacial Surgery, Jessenius Faculty of Medicine in Martin, Comenius University Bratislava, Kollárova 2, 03601 Martin, Slovakia; augustin24@uniba.sk; 2 University Hospital Martin, Kollárová 2, 03601 Martin, Slovakia

**Keywords:** digital diagnostics, artificial intelligence, near-infrared light transillumination, dental caries, minimally invasive dentistry, intelligence augmentation, clinical decision-making

## Abstract

Background: Approximal dental caries pose a persistent diagnostic challenge, as early lesions frequently remain clinically occult beneath intact enamel surfaces during routine visual–tactile inspection. This case report describes the clinical utility, limitations, and decision-making discrepancies within a multimodal digital diagnostic workflow combining intraoral radiography, near-infrared light transillumination (NILT), and deep-learning artificial intelligence (AI) radiographic analysis. Case Presentation: A 19-year-old female patient presented with provoked cold and sweet sensitivity localized to the upper left quadrant. Clinical inspection revealed visually intact accessible enamel surfaces (ICDAS 0 on tooth 25. To resolve the discrepancy between subjective symptoms and inconclusive initial visual findings, periapical radiography and NILT were performed. After the clinician-led treatment plan had been established, the radiograph was additionally processed using ClinicalAID as a parallel research analysis. Diagnostic Assessment and Management: Periapical radiography and NILT revealed approximal demineralization on teeth 24, 25, and 26. After the clinician-led treatment plan had been established, the radiograph was additionally processed with ClinicalAID as a parallel research analysis, yielding model confidence scores of 100%, 99%, and 98% for teeth 24, 25, and 26, respectively. Pre-operative therapeutic stratification remained entirely clinician-led: teeth 24 and 25 were selected for operative intervention based on provoked symptomatology and radiographic appearance of dentinal involvement, whereas tooth 26 was managed non-operatively with topical fluoridation and scheduled monitoring. Operative clinical reference assessment confirmed demineralization extending into the middle third of the dentin on tooth 25. Conclusions: High AI caries-detection confidence scores do not differentiate between lesion depth, cavitation status, or the necessity of operative intervention, as evidenced by divergent clinical pathways despite near-identical AI output scores on teeth 25 and 26. Deep-learning algorithms must function strictly as adjunctive decision-support tools within a human-in-the-loop framework (Intelligence Augmentation), with treatment planning governed by clinical symptoms, caries risk, and conventional diagnostic synthesis.

## 1. Introduction

In an era of significant digital advancement in medicine, the World Health Organization (WHO) continues to raise serious concerns regarding the high global prevalence of oral diseases, particularly dental caries. These conditions currently affect approximately 3.5 billion people worldwide [1]. Dental caries remains among the most widespread global health challenges across all age groups [2], with the Global Burden of Disease study identifying untreated permanent tooth decay as the most prevalent health condition of global significance [3].

Dental caries is currently recognized as a biofilm-mediated, diet- modulated, multifactorial, non-communicable, dynamic disease resulting in net mineral loss of dental hard tissues [4,5]. The caries process is dynamic, and early detection of non-cavitated lesions provides an opportunity for non-restorative interventions aimed at arresting or reversing lesion progression [6]. This paradigm shift from traditional, invasive cavity preparation toward the preventive management of non-cavitated lesions forms the core foundation of contemporary minimally invasive dentistry [7].

From a clinical standpoint, accurately differentiating between purely enamel-bound lesions and those extending into the dentin is essential for correct decision-making. Consequently, current research is heavily focused on developing innovative diagnostic modalities capable of identifying caries at its initial, non-cavitated stages [8]. Although conventional visual–tactile examination and intraoral radiography remain fundamental components of caries diagnosis, their diagnostic performance varies according to the diagnostic method, lesion characteristics, and clinical setting [9,10]. This variability underscores the need for supplementary diagnostic methods to improve the consistency and accuracy of proximal caries detection.

Advanced optical technologies, such as laser fluorescence and near-infrared light transillumination (NILT), offer a promising solution by allowing clinicians to visualize the spatial extent and optical characteristics of carious lesions [10]. Transillumination-based methods, such as the Near-Infrared Digital Imaging Caries Assistance (e.g., DIXION/DIAGNOcam technology), provide a crucial, non-invasive alternative to traditional radiographic methods that rely on ionizing radiation [11]. By eliminating radiation exposure, these technologies safely enable regular, longitudinal monitoring of borderline lesions over time. The most recent transformative trend in dental diagnostics involves the integration of artificial intelligence (AI), which is rapidly shifting oral radiology from a purely descriptive discipline into a highly predictive one [12]. Advanced AI algorithms, particularly convolutional neural networks (CNNs), have demonstrated promising diagnostic performance in the detection of dental caries and may serve as an adjunct to conventional clinician-based diagnosis [13,14]. From a health-economic perspective, AI-assisted caries detection has been discussed as having potential clinical and economic implications; however, its cost-effectiveness remains dependent on the specific diagnostic setting, downstream treatment decisions, and assumptions used in economic modelling [15,16]. Current evidence demonstrates high diagnostic accuracy for both NILT [10] and radiographic deep learning algorithms [13,14] when evaluated as isolated modalities. However, in routine clinical pathways, these technologies are rarely implemented in a unified, sequential diagnostic workflow where discordant signals between modalities must be resolved. Published evidence specifically addressing the combined use of near-infrared transillumination and commercial deep-learning radiographic analysis for supporting clinician-led treatment decision-making in visually intact approximal surfaces remains limited. This case report illustrates the diagnostic yield, discordances, and clinical decision-making within such an integrated workflow for clinically occult or visually inapparent approximal lesions.

## 2. Case Report

### 2.1. Patient Information and Clinical Findings

A 19-year-old female patient presented to a private dental clinic in Slovakia, complaining of persistent discomfort in the upper left quadrant (second quadrant). The pain was explicitly provoked by external chemical and thermal stimuli, particularly sweet and cold food/beverages, lasting for approximately three days. The patient’s personal medical, family, systemic medication, and allergy histories were unremarkable. The patient was classified as having a moderate caries risk. Oral hygiene was considered good, with an O’Leary Plaque Control Record of approximately 25%. She reported regular use of fluoridated toothpaste and a low frequency of fermentable carbohydrate intake.

**Figure 1:**
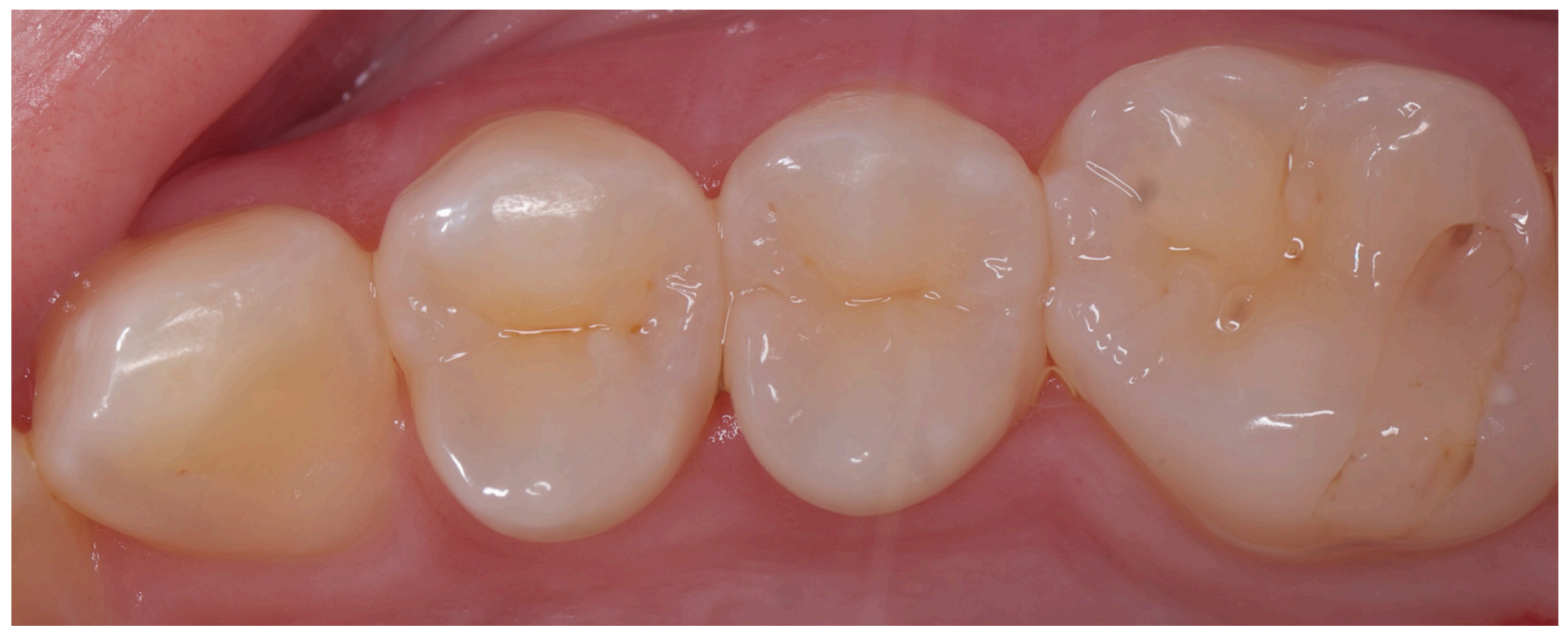
Initial photographic documentation of teeth 24, 25, and 26 in the second quadrant (Authors’ archive).

Extraoral clinical examination revealed normal soft tissue contours with no pathological findings or lymphadenopathy. Intraoral visual and tactile examination demonstrated healthy, fully functional dentition; teeth 23, 24, 25, 26, and 27 were vital and non-tender to vertical or horizontal percussion. Cold testing was performed using ROEKO Endo-Frost (COLTENE, Altstätten, Switzerland). The interproximal region between teeth 24 and 25 elicited a brief provoked response lasting approximately 3–4 s, which subsided immediately after removal of the stimulus. The teeth were considered vital, with a brief provoked response to cold and sweet stimuli that resolved rapidly after removal of the stimulus. No spontaneous pain, lingering thermal pain, or percussion tenderness was present. Based on the clinical findings, the pulpal diagnosis was normal pulp for teeth 23, 24, 25, 26, and 27. The apical diagnosis was normal apical tissues for all examined teeth. Following dental prophylaxis and thorough air-drying, visual inspection of the accessible occlusal and approximal enamel surfaces revealed no visible discoloration, structural breakdown, or cavitation. Tooth 25 was therefore classified as ICDAS Code 0 on visual examination (International Caries Detection and Assessment System). No cavitation was visible on the clinically accessible approximal enamel surfaces; however, because the contact area itself was not directly accessible for complete inspection, the absence of cavitation at the hidden contact point could not be definitively established pre-operatively (Figure 1).

### 2.2. Multimodal Diagnostic Workflow

To investigate the diagnostic discrepancy between subjective symptoms and inconclusive initial visual findings, diagnostic modalities were executed and evaluated according to the following sequential protocol:

1. Clinical and Vitality Examination: Visual–tactile assessment, air-drying, cold testing, and percussion testing.
2. Conventional Radiographic Acquisition and Interpretation: Targeted periapical radiography evaluated under standard viewing conditions.
3. Optical Assessment: Near-infrared light transillumination and fluorescence assessment using NILT (DIAGNOcam Vision Full HD).
4. Clinical Management Stratification: Synthesis of clinical symptomatology and imaging findings by the treating clinician to formulate the provisional therapeutic plan.
5. Parallel Research AI Analysis: Automated radiographic processing using ClinicalAID (version 0.7.2) for experimental evaluation.
6. Operative Intervention: Selective caries removal serving as an operative clinical reference assessment for the restored surfaces.

The ClinicalAID software was deployed strictly under a research and assessment protocol and was not CE-marked for autonomous diagnostic or therapeutic planning at the time of patient presentation. The software interface incorporated explicit advisory notices stating that the output is not intended as a definitive diagnostic directive. Clinical decision-making was established on clinical findings and conventional imaging; AI outputs served solely as adjunctive, parallel research observations.

**Figure 2:**
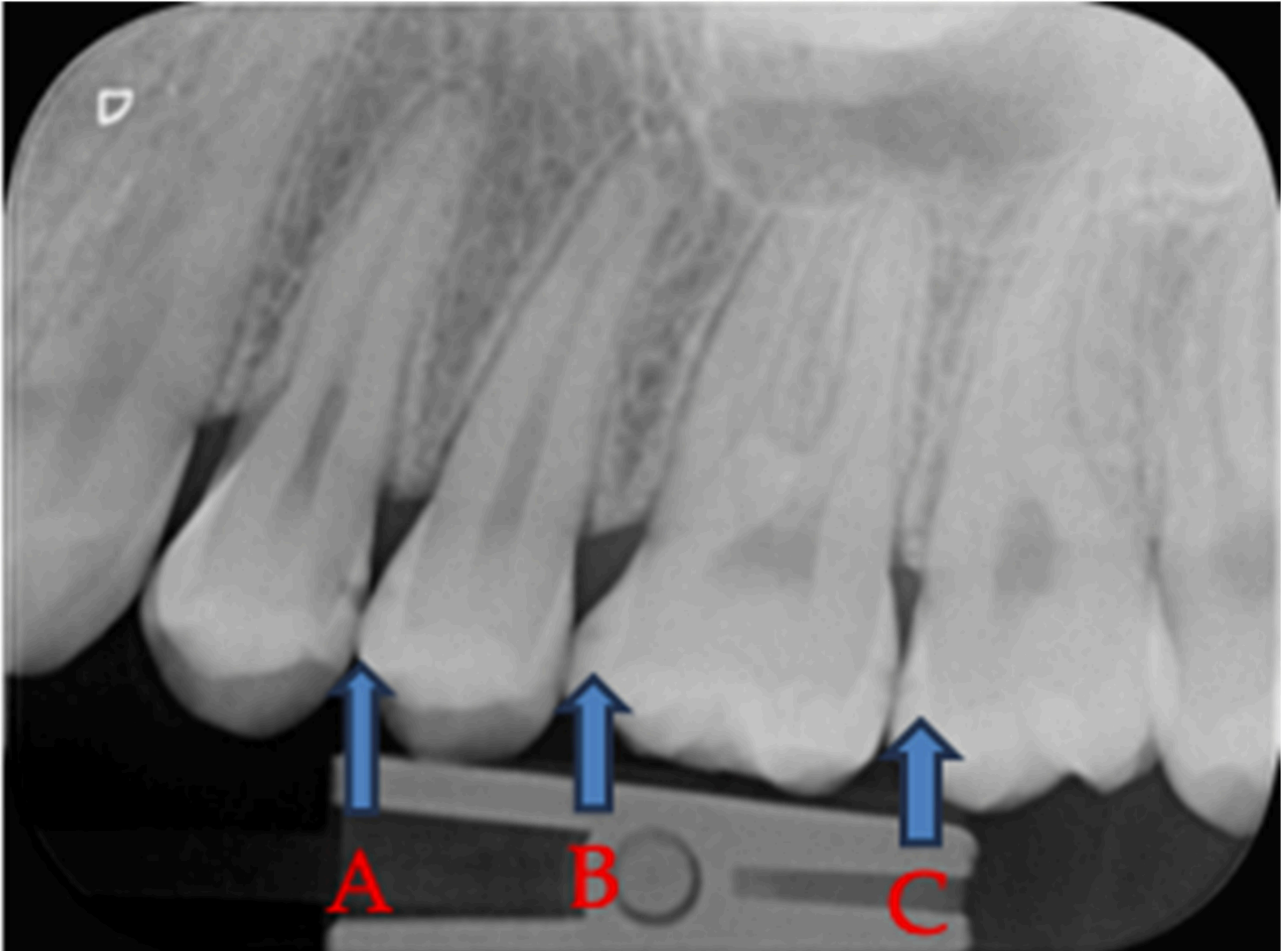
Periapical intraoral radiograph showing approximal radiolucencies: (A) distal surface of tooth 24 and mesial surface of tooth 25, with radiographic extension into dentin; (B) mesial surface of tooth 26; and (C) distal surface of tooth 26, with the lesion on tooth 26 predominantly confined to enamel (Authors’ archive).

Intraoral Radiography: A periapical projection was selected as part of the initial clinical assessment. Although it demonstrated the relevant approximal radiolucencies, the projection has limitations for caries-depth assessment compared with standardized bitewing radiography, particularly with regard to contact overlap and geometric distortion. A targeted periapical intraoral radiograph was obtained, revealing radiolucent lesions on the distal surface of tooth 24 and the mesial surface of tooth 25 extending into dentin, whereas the mesial and distal lesions on tooth 26 appeared predominantly confined to enamel (Figure 2).

Near-infrared light transillumination (NILT) and fluorescence imaging were performed using the DIAGNOcam Vision Full HD system (KaVo Dental GmbH, Biberach, Germany). The optical transillumination and fluorescence modalities were interpreted independently to prevent diagnostic conflation: transillumination was evaluated specifically for optical shadow formation indicative of mineral density loss, whereas fluorescence output was interpreted descriptively without inferring quantitative microbiological status.

**Figure 3:**
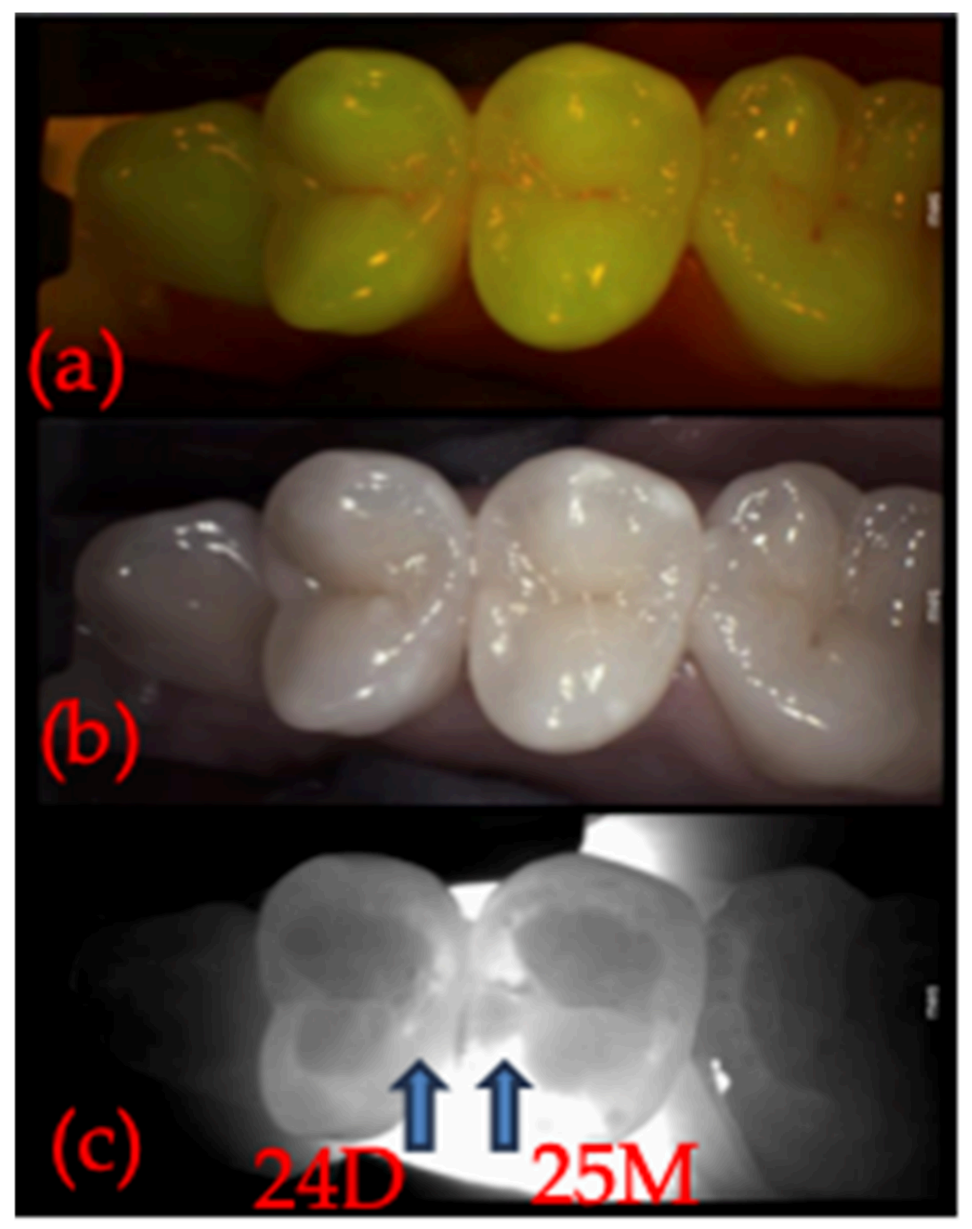
DIAGNOcam Vision Full HD diagnostic output focused on teeth 24 and 25: (**a**) fluorescence mode; (**b**) intraoral mode; (**c**) transillumination mode. The distal surface of tooth 24 (24D) shows a dark shadow extending beyond the enamel–dentin junction, compatible with dentinal involvement, whereas the mesial surface of tooth 25 (25M) shows a well-defined shadow localized at the enamel–dentin junction (Authors’ archive).

**Figure 4:**
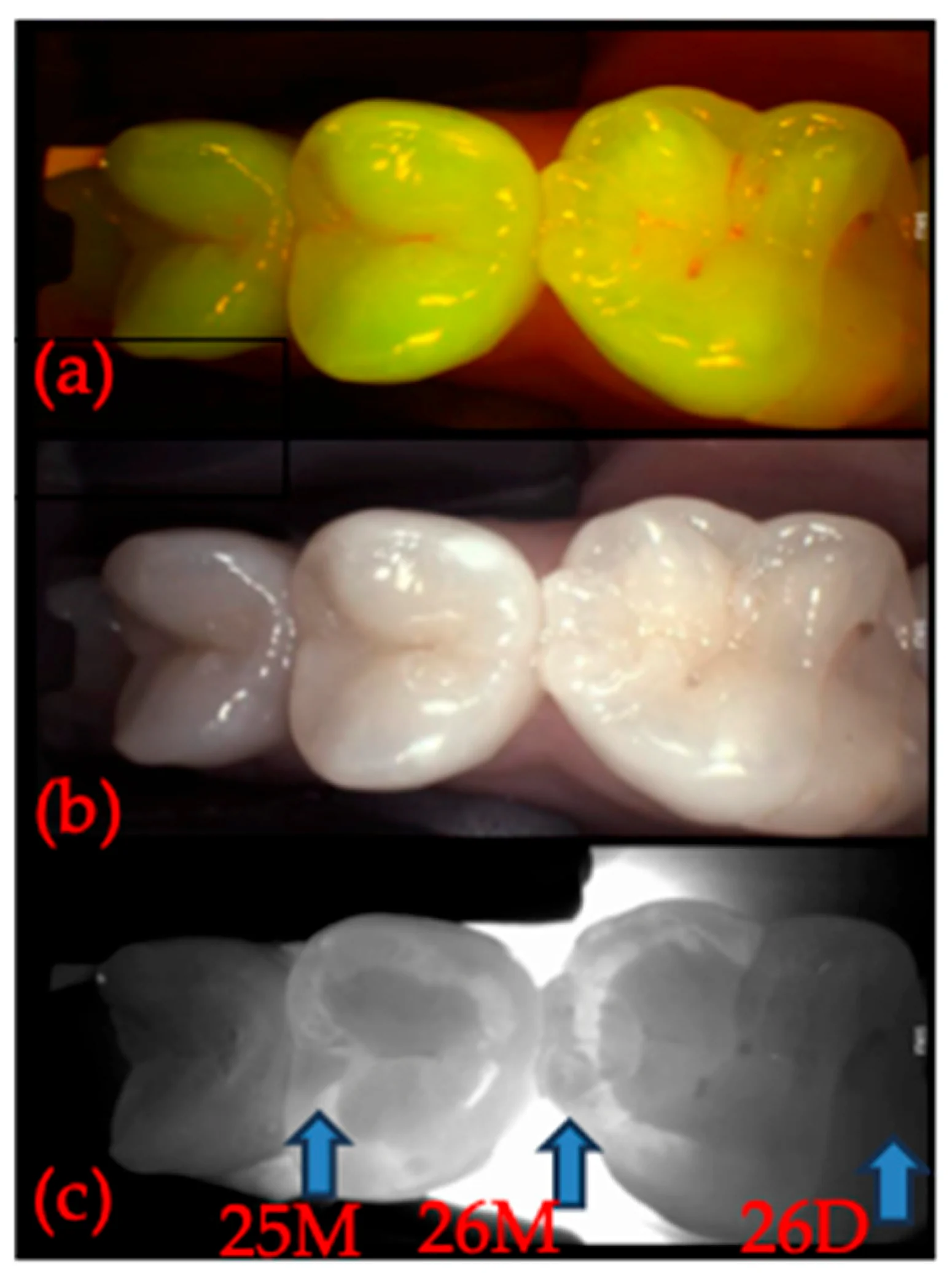
DIAGNOcam Vision Full HD diagnostic output documenting lesions on teeth 25 and 26: (**a**) fluorescence mode; (**b**) intraoral mode; (**c**) transillumination. The mesial surface of tooth 25 (25M) shows a well-defined shadow localized at the enamel–dentin junction, while the mesial and distal surfaces of tooth 26 (26M and 26D) show localized enamel-confined shadows (Authors’ archive).

Transillumination imaging demonstrated distinct optical characteristics across the affected sites:

- Tooth 24 (distal): An optical shadow extending past the enamel–dentin junction (EDJ) into the outer dentin, consistent with dentinal involvement (Figure 3).
- Tooth 25 (mesial): A well-demarcated approximal shadow localized at the enamel–dentin junction (EDJ), without discernible deep penetration toward the pulpal complex on transillumination alone (Figure 3 and Figure 4).
- Tooth 26 (mesial and distal): Discrete, superficial optical shadows confined strictly within the enamel layer (Figure 4).

Accompanying fluorescence acquisition demonstrated localized orange-red signal emission within the interproximal embrasure spaces between teeth 24–25 and 25–26. This finding was recorded descriptively as an additional fluorescence observation and was not interpreted as a quantitative measure of microbial activity, bacterial load, or caries lesion activity. To improve procedural consistency, all diagnostic procedures were performed under standardized clinical conditions by the same experienced operator. Radiographic acquisition, NILT imaging, and clinical examination were carried out using the same devices and routine acquisition settings throughout the case assessment. Image quality was assessed immediately after acquisition before interpretation. Despite these measures, multimodal diagnostic interpretation remains partly operator-dependent, and variations in acquisition technique, equipment characteristics, and examiner experience may influence diagnostic performance and limit the generalizability of the findings.

The intraoral periapical radiograph was processed post-acquisition using the deep-learning convolutional neural network software ClinicalAID (version 0.7.2, AID s.r.o., Bratislava, Slovakia). ClinicalAID processes raw PNG radiographic images to output annotated bounding boxes and tooth-specific model confidence scores. The software output yielded the following confidence scores: tooth 24 (1.00/100%), tooth 25 (0.99/99%), tooth 26 (0.98/98%), and tooth 27 (0.24/24%). Crucially, these numerical outputs represent proprietary model confidence scores reflecting the internal likelihood of lesion detection on 2D radiographs, rather than calibrated posterior probabilities of biological disease, lesion depth, or cavitation. Tooth 27 yielded a model confidence score of 24%, exceeding the software detection threshold of 0.07 (7%) and therefore representing a positive caries-lesion detection. A superficial enamel-confined radiolucency was observed radiographically on the mesial surface of tooth 27; NILT assessment of this tooth was not performed.

According to developer-supplied validation data (unpublished proprietary documentation, AID s.r.o.), the model was trained on 3416 dental radiographs (1380 panoramic and 2046 intraoral images) encompassing over 34,000 annotated tooth regions. The apparent inconsistency arises from the prevalence figure and the predictive values being derived from different datasets. The prevalence stated in the manufacturer’s IFU (11%) refers to the combined training and test data, whereas sensitivity, specificity, PPV, and NPV were computed on the independent test set, in which caries prevalence was 9.7% (604 positive regions of interest out of 6245). Recomputing the predictive values using a sensitivity of 0.785, a specificity of 0.944, and a prevalence of 9.7% yields a PPV of 0.600 and an NPV of 0.976, exactly matching the reported values. The discrepancy is therefore not attributable to rounding or to aggregation of regions of interest.

**Figure 5:**
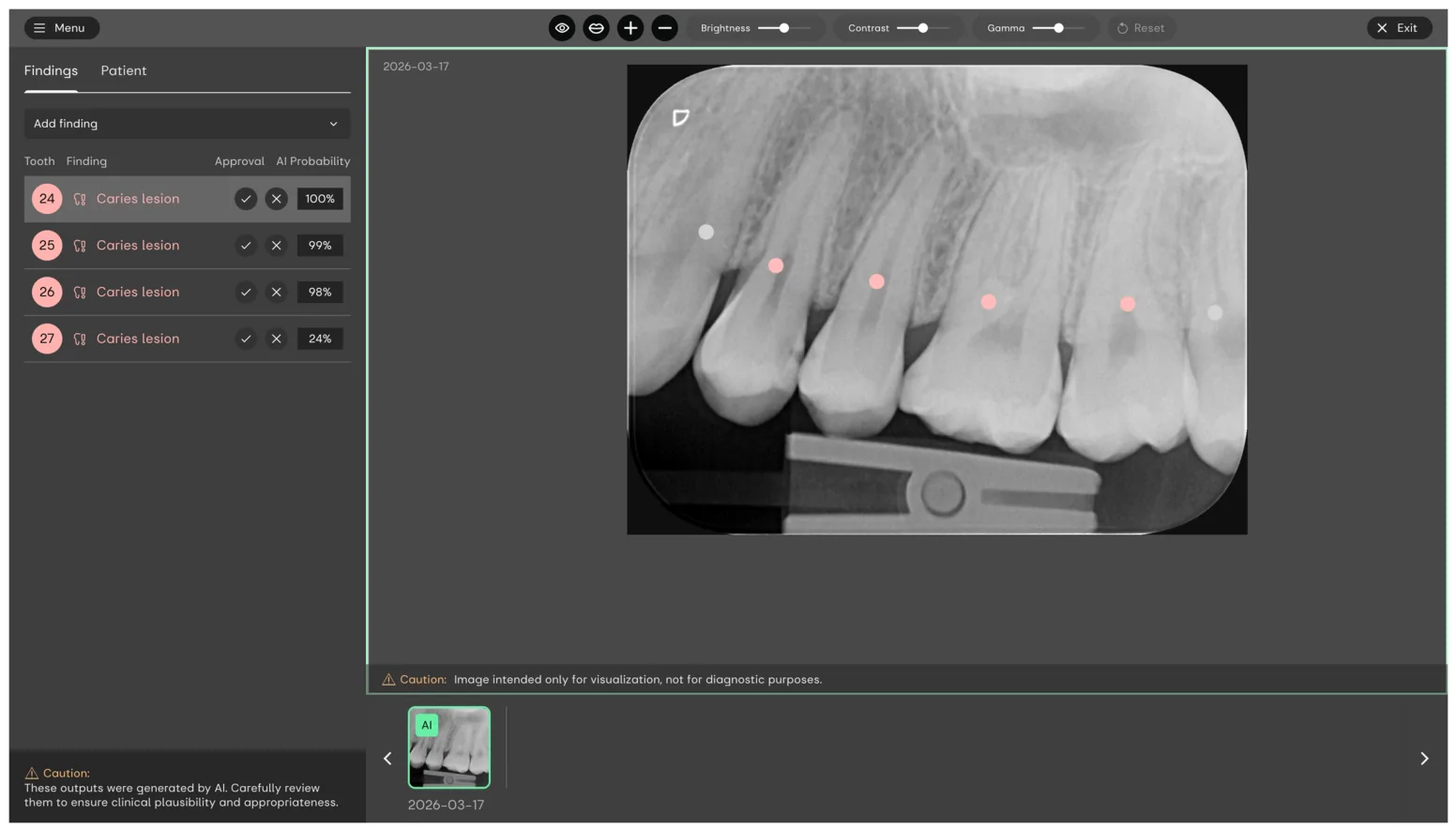
Representative ClinicalAID software output of the periapical intraoral radiograph showing AI-assisted caries detection and tooth-specific model confidence scores (Authors’ archive).

However, these metrics reflect agreement against an expert consensus radiographic reference standard rather than histological or operative ground truth. Furthermore, test metrics stratified specifically for periapical radiographs of approximal surfaces are not available, and general validation figures cannot be assumed to translate directly to isolated periapical projection scenarios. Further methodological details, including the test-set denominators, 2 × 2 contingency data, patient-level splitting, the distribution of bitewing versus periapical intraoral images, and version-specific validation results, were not available to the authors. Accordingly, the reported global validation metrics should be regarded as descriptive developer-supplied performance data and should not be interpreted as task-specific performance estimates for periapical detection of approximal caries in the present case. A representative ClinicalAID software output is shown in Figure 5.

The AI model output identified the following:

Tooth 24: Caries lesion, model confidence score 100%.

Tooth 25: Caries lesion, model confidence score 99%.

Tooth 26: Caries lesion, model confidence score 98%.

Tooth 27: Caries lesion, model confidence score 24%.

The software output did not provide treatment recommendations. The primary clinical management decisions were established by the clinician on the basis of clinical, radiographic, and NILT findings; the subsequent ClinicalAID analysis was documented as a parallel research assessment. A comparative summary of the diagnostic outputs across all modalities is presented in Table 1.

The 24% model confidence score recorded for tooth 27 exceeded the software detection threshold of 0.07 (7%) and was therefore reported as a positive caries-lesion detection. As the model output did not constitute a treatment recommendation, tooth 27 was managed non-operatively with topical fluoridation and clinical monitoring based on the integrated clinical and radiographic findings.

For tooth 25, the decision to proceed with restorative treatment was made pre-operatively by the clinician based on the clinical findings, including the provoked sensitivity originating from the 24–25 interproximal region, together with the radiographic appearance of the approximal lesion, which was clinically interpreted as extending into dentin, and the complementary NILT findings. The subsequent ClinicalAID analysis was performed after the primary treatment decision and was documented as a parallel research assessment; it did not influence the decision to restore tooth 25. The clinical timeline is summarized in Table 2.

### 2.3. Treatment Procedure

Resin infiltration was not selected for tooth 25 because the lesion was radiographically interpreted as extending into dentin and, in the context of provoked sensitivity originating from the 24–25 interproximal region, operative assessment and restorative management were considered more appropriate than a micro-invasive approach.

A combined therapeutic strategy was devised based on a synthesis of the multimodal findings. Local infiltration anesthesia was administered using Septanest (4% Articaine with 1:200,000 epinephrine; Septodont, Saint-Maur-des-Fossés, France), and complete isolation of the operating field was achieved using a rubber dam system.

**Figure 6:**
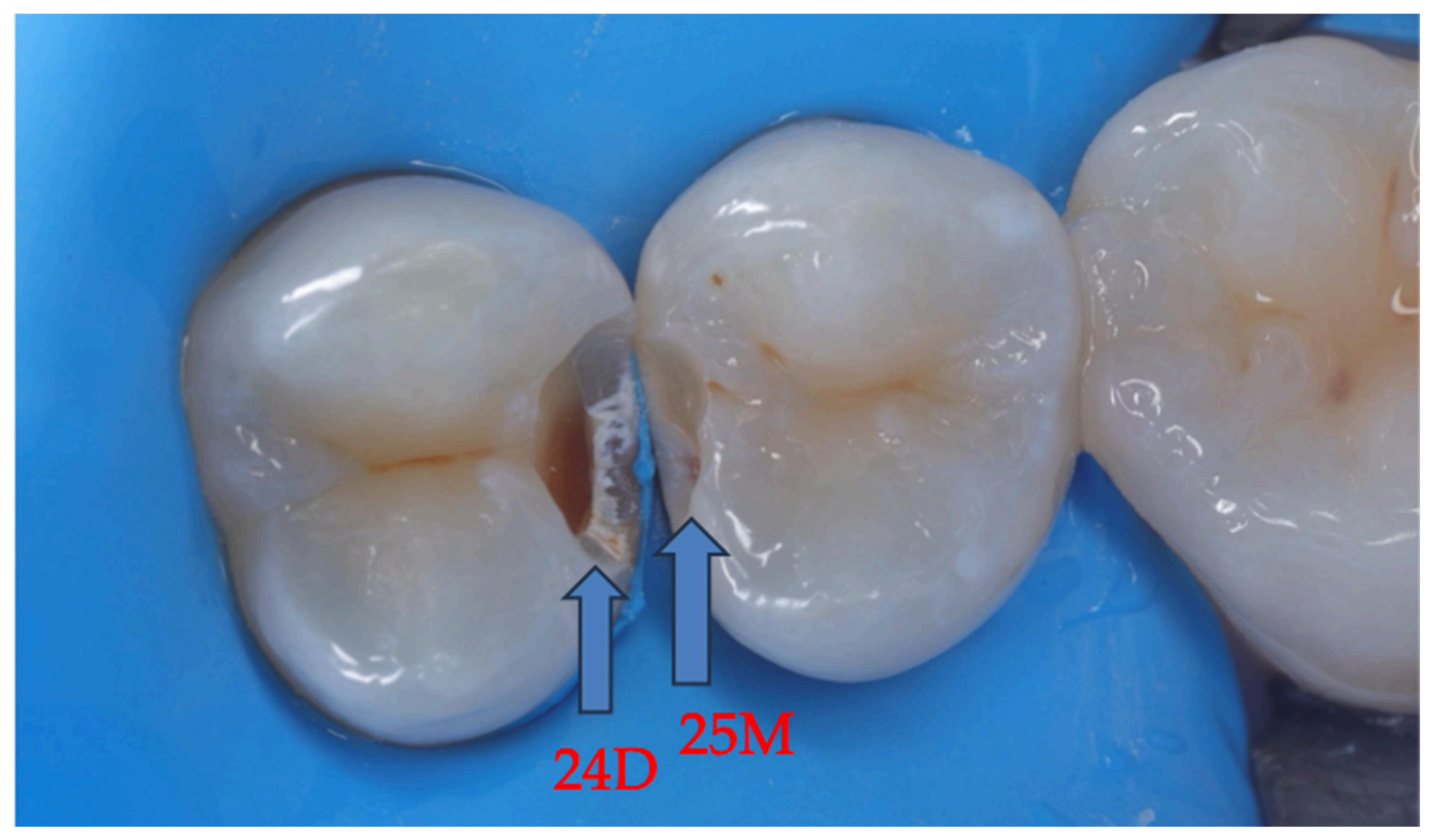
Clinical extent of the caries lesions on tooth 24 distal (24D) and tooth 25 mesial (25M) after operative access and selective tissue removal (Authors’ archive).

Upon conservative surgical access to the lesions, we observed the following:

- Tooth 24: Opening of the distal cavity confirmed primary caries extending through the enamel into peripheral dentin (Figure 6).
- Tooth 25: For tooth 25, operative access revealed demineralized dentin extending into the middle third.

Operative Clinical Reference Assessment: Direct visual–tactile inspection following operative access and selective carious tissue removal was used to assess dentinal involvement in teeth 24 and 25. Histological validation was not performed as it is clinically and ethically inapplicable in vital, restorable teeth.

Infected dentin was selectively excavated using low-speed rotary burs and hand instruments. At the peripheral cavity margins, carious tissue was removed to hard dentin to obtain sound enamel–dentin margins, whereas excavation toward the pulpal aspect was performed selectively to firm dentin. No pulp exposure or other intraoperative complications occurred. The cavities were etched with 37% phosphoric acid gel using ANA Etching Gel (Directa AB, Upplands Väsby, Sweden), treated with G-Premio BOND (GC Corporation, Tokyo, Japan), and restored using Neo Spectra™ ST LV A2 and Neo Spectra™ ST flow A2 composite materials (Dentsply Sirona, Konstanz, Germany) using an incremental layering technique. Final finishing and polishing were completed to restore anatomical contours and contact points.

### 2.4. Clinical Outcome and Follow-Up

At the 48-h post-operative evaluation, the patient reported complete resolution of cold and sweet sensitivity with no post-operative discomfort. Tooth 26 was managed non-invasively according to a preventive protocol including localized high-concentration fluoride varnish application using Duraphat (22 600 ppm F; Colgate-Palmolive, New York, NY, USA), interdental hygiene instruction, and scheduled digital/radiographic monitoring at a 6-month interval. At the time of manuscript preparation, no longer-term clinical or radiographic follow-up data were available; therefore, the reported outcome is limited to short-term symptom resolution and the planned preventive monitoring of tooth 26.

## 3. Discussion

The presented case report illustrates the integration and clinical interpretation of a multimodal digital diagnostic approach in modern restorative dentistry. The timely identification of approximal caries lesions that may remain visually inapparent during routine visual–tactile examination remains a challenge in daily clinical practice [7]. As highlighted by contemporary cariology frameworks, dental caries management has progressively shifted from routine invasive operative treatment toward longitudinal monitoring, non-operative preventive approaches, and micro-invasive strategies [5,6]. This paradigm shift increases the importance of diagnostic methods capable of identifying and characterizing lesions before extensive structural breakdown occurs [7,8].

In the present case, the clinically accessible surfaces appeared visually sound, whereas provoked sensitivity originating from the interproximal region between teeth 24 and 25 prompted further diagnostic assessment. Intraoral radiography remains an important modality for detecting approximal radiolucencies [10,17]; however, radiographic interpretation is susceptible to inter- and intra-observer variability, projection geometry, and contact overlap [9,10,17]. Deep-learning algorithms, particularly convolutional neural networks, have therefore been investigated as adjunctive tools for radiographic caries detection and image interpretation [13,14,18]. Nevertheless, AI-generated findings should be regarded as complementary information rather than as independent determinants of diagnosis or treatment.

A central observation of the present case is the marked difference in clinical management between teeth 25 and 26 despite their nearly identical numerical ClinicalAID outputs. ClinicalAID generated caries-detection outputs of 99% for tooth 25 and 98% for tooth 26. Tooth 25 underwent operative restorative treatment, whereas tooth 26 was selected for non-operative management with topical fluoride varnish and scheduled monitoring. The similarity of these numerical outputs despite substantially different management decisions illustrates that the magnitude of an AI-generated caries-detection output cannot, by itself, be interpreted as information regarding lesion depth, activity, surface cavitation, pulpal status, or restorative treatment need.

The clinician based treatment stratification on the clinical findings, radiographic interpretation, and NILT assessment. For tooth 25, the radiographic appearance was clinically interpreted as suggestive of dentinal involvement, while NILT demonstrated an optical shadow localized at the enamel–dentin junction. The provoked sensitivity contributed to the overall clinical and pulpal assessment; however, it could not be unequivocally attributed to tooth 25 because the response originated from the interproximal region between teeth 24 and 25. Tooth 24 also demonstrated dentinal caries, and both teeth were restored during the same appointment. Consequently, the subsequent resolution of sensitivity cannot establish which lesion was primarily responsible for the symptoms. Importantly, provoked sensitivity should not be interpreted as a direct indicator of caries lesion activity. The subsequent ClinicalAID analysis was performed only after the primary treatment decision had been established and was documented as a parallel research assessment.

Consequently, deep-learning caries-detection outputs should be interpreted cautiously and should not be regarded as calibrated probabilities of disease, lesion severity, or treatment need unless formal calibration has been demonstrated. In the present case, the ClinicalAID output represented a model-generated caries-detection score rather than an assessment of lesion depth, activity, cavitation, or treatment threshold [19,20]. Clinical determination of whether a lesion warrants non-operative monitoring, micro-invasive management, or operative restoration requires comprehensive clinical synthesis, including surface integrity, radiographic lesion extent, pulpal status, individual caries risk, symptomatology, patient-specific factors, and feasibility of longitudinal monitoring. Such decisions remain within the remit of clinician-led judgement under the framework of Intelligence Augmentation [19,20].

The diagnostic performance of AI-based caries detection should also be interpreted in relation to lesion type and imaging modality. Previous systematic reviews have demonstrated considerable heterogeneity in reported AI performance across intraoral photographs, periapical and bitewing radiographs, near-infrared transillumination, optical coherence tomography, and panoramic imaging [13]. Moreover, diagnostic sensitivity may differ according to lesion depth; a recent systematic review and meta-analysis of AI-assisted caries detection on bitewing radiographs reported higher sensitivity for dentinal caries than for enamel caries [21]. Therefore, performance estimates obtained for one lesion type or imaging modality cannot necessarily be extrapolated directly to other clinical scenarios. In the present case, ClinicalAID was applied exclusively to a periapical radiograph for the assessment of approximal lesions; accordingly, its output should be interpreted within this specific lesion and imaging context [21].

Near-infrared light transillumination (NILT; DIAGNOcam) provided complementary non-ionizing optical visualization of the affected approximal regions. Transillumination demonstrated an optical shadow localized at the enamel–dentin junction on tooth 25 and predominantly enamel-confined shadows on tooth 26 [10,11]. Operative access to tooth 25 subsequently demonstrated demineralized dentin extending into the middle third. Because no blinded or quantitative comparison between NILT appearance and operative lesion extent was performed, this difference should not be interpreted as evidence that NILT underestimated lesion depth. Rather, the observations demonstrate that optical shadow localization and operative lesion extent represent different forms of diagnostic information.

Previous studies have investigated the diagnostic performance of NILT and other optical modalities, including optical coherence tomography, for caries detection [22,23]. However, lesion progression over time was not assessed in the present case, and optical shadow depth was not validated against an independent reference standard. NILT should therefore be regarded as a complementary optical modality for lesion detection and visualization rather than as an independent quantitative measure of three-dimensional lesion depth. Transillumination and fluorescence outputs were interpreted separately. The orange-red fluorescence observed in the embrasure spaces was recorded descriptively as an additional fluorescence finding and was not interpreted as a quantitative measure of microbial activity, bacterial load, or lesion activity.

The operative visual–tactile assessment performed after cavity access served as a pragmatic clinical reference assessment for teeth 24 and 25. It should not, however, be considered an unbiased “ground truth”. Operative verification was available only for teeth selected for restorative treatment, whereas tooth 26, which was managed non-operatively, did not undergo the same reference assessment. This introduces partial verification, or work-up, bias. Moreover, the operative assessment was performed within a sequential clinical workflow in which the clinician was aware of the preceding clinical and imaging findings. Concordance between modalities therefore cannot be interpreted as independent cross-validation.

From a broader perspective, digital diagnostic aids and longitudinal image archiving have been discussed in health-economic models as potentially cost-effective strategies for reducing long-term restorative burdens [15,16]. However, economic outcomes depend strongly on clinical decision thresholds and the avoidance of overtreatment, and no conclusions regarding cost-effectiveness can be drawn from a single clinical case [15,16]. As emerging digital tools continue to enter clinical workflows, dental education and regulatory frameworks must evolve to ensure that clinicians understand algorithmic limitations, avoid automation bias, and apply trustworthy, human-centered approaches to AI-assisted care [24].

Dental curricula must similarly adapt so that future clinicians understand both the capabilities and the limitations of AI tools and are able to critically interpret AI-generated outputs within the complete clinical context [25].

Overall, the current literature supports the use of artificial intelligence as an adjunctive tool in dental diagnostics, with potential benefits in image interpretation and workflow support when integrated with conventional assessment [12,26]. Such approaches should be implemented within a clinician-supervised, human-in-the-loop framework rather than as autonomous diagnostic systems. In the present case, however, the ClinicalAID analysis was performed only after the primary clinician-led treatment decision had already been established. The AI output was therefore documented as a parallel research assessment and did not determine the treatment decision [12,26].

Previous studies evaluating AI-assisted caries detection have predominantly focused on the diagnostic performance of AI systems applied to radiographic images, often by comparing AI outputs with expert assessment or conventional reference standards [21,27]. In contrast, the present case did not evaluate ClinicalAID as a stand-alone diagnostic system. Its distinctive feature was the sequential clinical workflow comprising clinical examination, symptom assessment, periapical radiography, NILT, clinician-led treatment stratification, and subsequent parallel AI analysis followed by operative reference assessment of the restored teeth. This case therefore illustrates how an AI-generated caries-detection output may be examined alongside a broader clinical reasoning process without being regarded as an autonomous determinant of treatment.

The different management of teeth 25 and 26 is particularly informative in this context. Despite similarly high AI-generated caries-detection outputs, the two teeth presented different clinical and imaging scenarios and were managed differently. This observation emphasizes that a high caries-detection output should not be regarded as a surrogate for lesion depth, cavitation, activity, or treatment need. In particular, the present findings do not demonstrate that the AI output validated or confirmed the treatment decision. Rather, they illustrate why numerical AI outputs require interpretation within the complete clinical context.

Tooth 26 was selected for non-operative management based on the available clinical and imaging findings. However, no long-term clinical or radiographic follow-up was available at the time of manuscript preparation. Consequently, the present report does not demonstrate remineralization, lesion arrest, or successful preventive treatment of tooth 26; it documents only the clinician’s decision for non-operative management and planned longitudinal monitoring.

Several methodological limitations of the present report must be acknowledged. Diagnostic modalities were evaluated sequentially by the treating clinical team rather than independently and under blinded conditions; therefore, agreement between methods cannot be interpreted as independent diagnostic confirmation. The operative clinical reference assessment was performed only on teeth undergoing restorative preparation, introducing partial verification bias. The clinician performing the operative assessment was aware of the preceding clinical and imaging findings. Furthermore, no long-term follow-up was available for tooth 26. As an isolated case report, the present study does not permit determination of diagnostic sensitivity, specificity, predictive values, clinical effectiveness, cost-effectiveness, or generalizability of the evaluated technologies. These questions require appropriately designed prospective investigations using standardized imaging protocols, independent reference assessments, and modality-specific validation [10,14].

## 4. Conclusions

This case report illustrates the integration and clinical interpretation of complementary diagnostic modalities in the assessment of clinically occult or visually inapparent approximal caries lesions.

Conventional intraoral radiography and near-infrared light transillumination (NILT) provided complementary diagnostic information in this case. The subsequent deep-learning analysis provided an additional research-level radiographic output but did not contribute to the primary treatment decision. This multimodal diagnostic approach may support the assessment of clinically occult or visually inapparent approximal lesions while preserving clinician-led diagnostic synthesis and treatment planning.

Artificial intelligence algorithms may serve as adjunctive analytical tools providing additional information for clinician-led decision-making. When used as an adjunctive analytical tool, AI may provide additional information for interpretation alongside clinician assessment. Furthermore, the combined interpretation of complementary digital modalities may assist in characterizing lesions with different clinical presentations and management pathways, as illustrated by the decision to manage tooth 26 non-operatively and teeth 24 and 25 operatively.

Critically, this case underscores that while digital and AI-driven diagnostic tools may provide useful adjunctive analytical support, final diagnostic synthesis and treatment authorization must remain under direct human clinical oversight within the framework of Intelligence Augmentation (IA). In this case, the decision to restore tooth 25 was established by the treating clinician on the basis of the clinical findings, provoked symptomatology, radiographic interpretation, and complementary NILT assessment. The subsequent ClinicalAID analysis was performed as a parallel research assessment and did not influence the primary treatment decision.

The integration of multimodal digital diagnostic approaches into routine dental practice may provide additional information that supports more consistent clinical decision-making, while their clinical value should be interpreted within the limitations of the available evidence.

## Figures and Tables

**Table 1:** Multimodal diagnostic comparison and integrated clinical decision-making.

Tooth/Surface	Visual/ICDAS	Radiographic Finding	NILT (DIAGNOcam)	AI Software Output	Final Clinical Decision
24 distal	ICDAS 0	Radiolucency in dentin	Dark shadow in outer dentin	Caries lesion; Model confidence score 100%	Restorative Therapy
25 mesial	ICDAS 0	Radiolucency in dentin	Shadow at enamel–dentin junction	Caries lesion; Model confidence score 99%	Restorative Therapy
26 mesial and distal	ICDAS 0	Radiolucency in enamel	Shadow in enamel	Caries lesion; Model confidence score 98%	Non-invasive Fluoridation and Monitoring
27 mesial	ICDAS 0	Radiolucency in enamel	Not Assessed	Caries lesion; Model confidence score 24%	Non-invasive Fluoridation and Monitoring

**Table 2:** Timeline of clinical events, diagnostic workflow, and therapeutic interventions (CARE Guidelines).

Timeframe/ Sequence	Event/ ClinicalStage	Specific Findings & Procedures	Clinical Objective/ Impact
Day −3 to Day 0	Symptom Onset & Baseline Presentation	19-year-old female presents with provoked cold/ sweet sensitivity in quadrant II; medical history non-contributory.	Initial triage and Chief complaint recording.
Day 0 (Initial Assessment)	Visual & Clinical Examination	Sound accessible enamel surfaces (ICDAS 0 on 25); brief cold response (3–4 s) localized to 24–25 interproximal space; non-tender to percussion.	Established pulpal and apical status (normal pulp; normal apical tissues) and localized the diagnostic discrepancy.
Day 0 (+15 min)	Conventional Radiography	Periapical projection reveals approximal radiolucencies in dentin (teeth 24, 25) and enamel (tooth 26).	Primary radiological identification of internaltissue demineralization.
Day 0 (+30 min)	Optical Assessment (NILT)	DIAGNOcam Vision Full HD demonstrates optical shadow at EDJ (tooth 25) and enamel-confined shadows (tooth 26); non-specific embrasure fluorescence.	Radiation-free optical visualization of lesion-associated shadows at the EDJ and within enamel
Day 0 (+45 min)	Clinician- Led Treatment Stratification	Clinician synthesizes provoked symptoms and radiographic dentin extension: indicates operative restoration for 24 & 25, non-operative management for 26.	Primary therapeutic decision established independently by treating clinician.
Day 0 (+50 min)	Parallel Research AI Analysis	Intraoral PNG processed with ClinicalAID v0.7.2: Confidence scores: 1.00 (24), 0.99 (25), 0.98 (26), 0.24 (27).	Parallel research image-analysis evaluation, performed after the primary treatment decision.
Day 0 (+60 min to +120 min)	Operative Intervention & Reference Assessment	Rubber dam isolation; selective excavation to firm dentin pulpo-axially and hard dentin peripherally; composite restoration (Neo Spectra ST); fluoride varnish (Duraphat) on 26.	Operative clinical Reference assessment confirms demineralization into middle third of dentin (25); immediate defect restoration.
Day 2 (+48 h)	Short-Term Evaluation	Clinical reassessment; complete resolution of provoked cold and sweet sensitivity; uneventful healing.	Verification of short-term clinical success.
Month 6 (Scheduled)	Longitudinal Follow-Up	Scheduled digital transillumination (NILT) and bite-wing radiographic surveillance of tooth 26; oral hygiene review.	Longitudinal monitoring of non-operatively managed approximal enamel lesions.

## Data Availability

All relevant data supporting the findings of this case report are included within the article. Due to patient privacy and ethical restrictions protecting personal medical confidentiality, any further non-identifiable clinical records are available from the corresponding author upon reasonable request.
